# Microbial Musings – May 2020

**DOI:** 10.1099/mic.0.000942

**Published:** 2020-05-29

**Authors:** Gavin H. Thomas

**Affiliations:** ^1^​ Department of Biology, University of York, York, UK

**Keywords:** Microbial

As this extraordinary year continues, we are now seeing the first opportunities for researchers at universities and research institutes to get back into the laboratory. This is no return to normality however, as much planning in how labs will operate in a background of the continuing pandemic mean that even if we do choose to come back to the lab, we will be working in a highly constrained research environment. More than ever we need to be thoughtful of our colleagues and their circumstances and cooperate on unprecedented levels to try and do productive research with limited time, personnel and facilities available.

In this month we focus on microbes living in some more unusual hosts, from turtles to ducks, but we start first in the home of the Magpies. Our newest Microbe Profile is included in the May issue and is for the well-known bacterium *Bacillus subtilis* [1]. Written by Jeff Errington FRS (@Errington_Lab) and Lizah van der Aart (@LizahvdA) from the Centre for Bacterial Cell Biology (CBCB) in Newcastle, UK, the profile starts with an excellent graphical abstract prepared by Dr van der Aart that outlines the many reasons why this Gram-positive bacterium is such a powerful tool to understand microbial function and has so many useful industrial applications. *B. subtilis* shares with its Gram-negative equivalent *Escherichia coli* K-12 an ease and speed of growth that makes it highly amenable to experimental study. Unlike *E. coli,* however, the organism is naturally transformable and will readily recombine introduced DNA onto the chromosome. This makes it highly genetically tractable and the 168 strain has emerged as the model lab strain used around the world. In the early days of molecular microbiology this was the key organism for elucidating the genetics and biochemistry of the process of spore formation, increasing our fundamental understanding of cellular development and differentiation. Like *E. coli *, *B. subtilis* also has been used for the benefit of mankind for the production of proteins such as proteases and lipases used in a range of industrial applications and also a range of small molecules and natural products [2], an area worked on for many decades by Errington’s Newcastle colleague Colin Harwood (@harwood_colin) with researchers over the EU and beyond. The authors highlight open questions where they consider *B. subtilis* can continue to provide answers to new questions about bacterial cell biology, physiology and genetics. Bacteriologists from the 'next generation’ like Nicola Stanley Wall (@bacteriacities) in Dundee and Akos Kovacs (@EvolvedBiofilm) at DTU, Denmark, have done much to develop *B. subtilis* as a model system for colony biofilms, while others, such as *Microbiology* editors Emma Denham (@Gingermicrobe) in Bath, elucidating the roles of small RNAs in cellular function, and Henrick Strahl, (@HenrikStrahl) also at CBCB in Newcastle, using *B. subtilis* to learn more about cell-envelope function [3], are pushing forward our understanding of biology using this bacterium.

Sticking with the tenuous avian theme, we turn to a paper about a species of bacteria, *Riemerella anatipestifer *, which sits within the Flavobacteriaceae, and is a pathogen of domestic ducks, geese and turkeys amongst other birds. The paper from the group of Qinghai Hu from the Shanghai Veterinary Research Institute, PR China, examines the role of iron acquisition in the virulence of this pathogen [4]. Like many fastidious animal pathogens, it requires blood agar to be cultured and can be found in the blood of infected ducks, so the authors asked the question of whether haemolysin activity, as a route to release haemoglobin, thence iron, correlated with virulence. The authors find that on duck-blood agar-plate assays about 50 % of the strains from their large set of different serotypes had secreted haemolysin activity and in a direct assay of pore formation with duck erythrocyte extracts from all the strains had activity. They conclude that while this could be one route for iron acquisition in the bird, it is not the defining feature and that the variable lethal dose to ducks seen across these strains is not correlated with haemolytic activity.

Our second ‘exotic’ paper this month is the microbiome of a turtle, Krefft’s river turtle in fact, which is indigenous to Australia. In the work by Donald McKnight (@donaldmcknight2) and colleagues from James Cook University, Queensland, Australia, the authors determine the microbiome of various sites in and on the turtle, finding that *Proteobacteria* and *Bacteroidetes* were the most common phyla [5]. Interestingly they also sample the shell of the turtle, which is known to harbour its own unique microbiome of macroalgae and found that the bacterial communities were different in the areas that contained or lacked the algae. Interestingly in regions lacking algae they found *Nostocaceae* cyanobacteria, suggesting that they compete with the algae for the shell surface.

The next paper from this month is from the group of Dipankar Nandi at the Indian Institute of Science, Bangalore, India. They have been studying the function of a protein involved in the cold-shock response in *Salmonella enterica* subsp. Typhimurium [6]. In my earlier days I had a rather simplistic understanding of this response in bacterial cells, thinking mainly with my protein biochemist ‘hat’ on that enzymes would work slower, such that everything just slows down a bit. I think this was mainly as the opposite response, that of heat shock, was where many proteins involved in chaperone function and repairing misfolded proteins were discovered, so that temperature shock had a very ‘protein-focussed’ view. However, this was, of course, just due to my ignorance and not thinking about how temperature impacts on the other major types of macromolecules in the cell. An excellent review on bacterial adaptation to cold was published by Cecilia Arraiano and colleagues in *Microbiology* a few years ago [7] and gives an overview of the diverse changes to cellular physiology that occur during a temperature drop. For example, the lower temperature reduces membrane fluidity, impacting on membrane processes and bacteria respond by increasing the level of unsaturation in their fatty-acid chains, both in the inner membrane and outer membrane. An area where cold shock has provided broader fundamental insight is in how RNA responds to the temperature decrease, as important structural features of RNA that form at 37 °C are now swamped by all kinds of additional folding that occurs at lower temperatures. Hence important work on the cold-shock response genes, from groups such as Masayori Inouye at Rutgers University, USA, have revealed great insight into RNA metabolism and repair. One of the cold-shock response proteins (Csp), CspE, is the focus of the paper in this month’s edition, investigating its role in biofilm formation in *Salmonella enterica* serovar Typhimurium. CspE is a protein from the same family as CspA – the major Csp in *E. coli* and *Salmonella enterica* serovar Typhimurium – which both function as RNA chaperones to enable transcription and translation during cold shock [8]. Deletion of *cspE* results in loss of biofilm formation, which can be complemented by functional *cspE*. CspA represses biofilm formation and the data suggests that CspE influences levels of *cspA* expression, something already demonstrated by Inouye’s group in *E. coli* [9], such that its deletion results in high levels of CspA. The authors also examine the role of the two proteins in swarming motility and find the CspE is essential for this process. Hence, they conclude that different Csp proteins play overlapping but distinct roles of two different biological processes.

Another strong paper from Michael Dunn’s group at the Universidad Nacional Autónoma de México, Cuernavaca, Mexico, features in this issue, which is in fact the second from this group in 2020 (I might have to think of some kind of a prize) [10, 11]. Continuing the theme of studying nitrogen metabolism in nitrogen-fixing bacteria, in this paper his group identify the arginase enzyme from *Sinorhizobium meliloti,* which is required for use of l-arginine as the sole nitrogen source by first breaking it down to l-ornithine and urea. Expression of the gene encoding the arginase is induced by the presence of l-arginine in the growth media, which they demonstrate requires a genetically linked transcription factor, which binds to the promoter region of the arginase gene. Their group are now investigating whether the function of this pathway is relevant not just in free-living conditions but also in the symbiotic state.

Finally, our editor’s choice this month, selected by Senior editor Jen Cavet, from Manchester, UK, is a paper from Nuno Empadinhas and colleagues at the University of Ciombra, Portugal, characterizing the growth and survival of an unusual thermophilic Mycobacterium and one of its highly thermostable proteins [12]. Check out the Microbe Post blog for more details. The paper is also dedicated to the memory of Milton S. da Costa (1948–2020), the long-time Chair of microbiology at Coimbra, who was well known across the EU for his work on extremophiles, having been President of FEMS from 2007 to 2010, a co-founder of the European Academy of Microbiology and a frequent author of papers in Microbiology Society journals [13].

That’s it for May. Stay safe.

Gavin Thomas

Department of Biology, University of York, UK, YO10 5YW.
